# Evaluation of ChatGPT-5.2 responses to frequently asked questions about benign bone tumors

**DOI:** 10.3389/fonc.2026.1826346

**Published:** 2026-05-20

**Authors:** Batuhan Ayhan, Samet Batuhan Yoğurt, Zeliha Deniz Ayhan

**Affiliations:** 1 Department of Orthopedics and Traumatology, Haymana State Hospital, Ankara, Türkiye; 2 Department of Orthopedics and Traumatology, Kahta State Hospital, Adıyaman, Türkiye; 3 Department of Pathology, Faculty of Medicine, Gazi University, Ankara, Türkiye

**Keywords:** artificial intelligence, benign bone tumors, ChatGPT, orthopedic oncology, patient education

## Abstract

**Objective:**

Patients increasingly seek health-related information through artificial intelligence (AI)-based chatbots. However, the reliability and clinical quality of chatbot-generated patient information remain uncertain. This study aimed to evaluate the quality and reliability of chatbot-generated responses to frequently asked patient questions regarding benign bone tumors using a structured assessment model.

**Methods:**

This descriptive and methodological study comprised twenty patient-centered commonly asked questions formulated by three fellowship-trained orthopedic oncology specialists. The inquiries encompassed diagnosis, treatment, complications, follow-up, and lifestyle-related issues pertaining to prevalent benign bone tumors. The responses produced by ChatGPT-5.2 were assessed separately by three independent orthopedic oncology specialists who had no role in formulating the questions. The quality of the response was evaluated using the Quality Analysis of Medical Artificial Intelligence (QAMAI) methodology, encompassing accuracy, clarity, relevance, completeness, citation of sources and references, and utility. Each parameter was evaluated using a five-point Likert scale. The intraclass correlation coefficient (ICC) was employed to assess interobserver reliability.

**Results:**

The greatest scores were observed in accuracy (mean score 4.27), but completeness (3.19) and the provision of sources and references (3.03) displayed somewhat lower values. The overall QAMAI score was 21.39 out of 30, reflecting good response quality consistent with the validated scoring range of 18–23 points. Interobserver agreement demonstrated good reliability for total QAMAI scores (ICC = 0.84; 95% CI: 0.74–0.91). The subdomain ICC values ranged from moderate to good agreement.

**Conclusion:**

Chatbot-generated responses provide accurate and useful preliminary information on benign bone tumors. However, shortcomings in completeness and reliance on evidence-based citations indicate that chatbot outputs should be employed under the oversight of a physician. AI chatbots can aid in patient education but cannot replace clinical decision-making processes.

## 1 Introduction

Benign bone tumors are among the frequently encountered lesions in orthopedics and traumatology outpatient clinics (1). They are often detected incidentally. This situation worries patients and leads to a need for information (2). Lesions such as enchondroma, osteochondroma, osteoid osteoma, simple bone cyst, and non-ossifying fibroma, although generally benign, are most frequently questioned by patients regarding the risk of malignant transformation, the possibility of pathological fracture, the need for surgery, and their impact on daily life activities (1–5). This situation highlights the need for accurate, understandable, and standardized information in patient-physician consultations.

Patients are increasingly accessing health-related information through artificial intelligence (AI) applications and online resources (6, 7). A study showed that approximately 75% of internet users search for health-related information online (8). With the introduction of AI chatbots, this has transformed into a new method of information searching, replacing traditional internet searches. ChatGPT 4.0 alone is estimated to have 300 million weekly active users and 3.8 billion monthly visitors as of November 2024 (9). However, the accuracy, clarity, and clinical reliability of the information presented in these resources can vary significantly, and incomplete or inaccurate information can increase patient anxiety and lead to unnecessary tests and treatments (10). Therefore, it is crucial that patient information content is not only scientifically accurate but also clear, comprehensive, and usable in clinical practice (11).

Beyond model capability, the architecture of the input prompt is itself a critical determinant of AI-generated response quality. Meskó has emphasized that prompt engineering, the deliberate framing of input queries with respect to specificity, role assignment, and output structure, should be regarded as an emerging core competency in healthcare, since the same model can produce substantively different outputs depending on how a question is posed (12). Hsieh et al. further confirmed that structured prompts can yield significant performance gains in earlier-generation models on medical questions (13). Because real-world patient queries are typically submitted as plain, unstructured questions, evaluations relying on engineered prompts may overestimate the quality of information patients actually receive, a consideration that directly motivated the prompting protocol used in the present study.

In recent years, structured approaches have been developed to objectively assess the quality of medical responses. The Quality Analysis of Medical Artificial Intelligence (QAMAI) framework allows for a holistic evaluation of medical responses based on key criteria such as accuracy, clarity, relevance, comprehensiveness, resource-basedness, and usability (14). However, studies in the literature that systematically formulate patient-centered frequently asked questions about benign bone tumors and evaluate the responses using structured quality measures are limited. The aim of this study is to identify patient-centered frequently asked questions about benign bone tumors and to analyze the quality of responses using a QAMAI-based evaluation system.

## 2 Materials and methods

This study is a descriptive and methodological study aimed at evaluating the quality of responses to patient-focused frequently asked questions regarding benign bone tumors. The frequently asked questions were developed by three fellowship-trained orthopedic oncology surgeons, each with a minimum of five years of clinical experience in orthopedic oncology, working in tertiary care referral centers. The questionnaire was designed to cover the most common benign bone tumors encountered in clinical practice, reflecting the concerns most frequently raised by patients during outpatient consultations. The questions were systematically distributed across five thematic domains — diagnosis, treatment, follow-up, complications, and daily living activities — to ensure comprehensive topical coverage (**Table 1**).

**Table 1:** Frequently asked patient questions about benign bone tumors.

No	Question	Thematic domain
1	Is this mass cancerous?	Diagnosis
2	Why did this form? Is it my fault?	Diagnosis
3	Does imaging (X-ray/MRI) provide a definitive diagnosis?	Diagnosis
4	Is having pain a bad sign?	Diagnosis
5	Does a biopsy cause the tumor to spread or worsen?	Complications
6	Is surgery mandatory for me?	Treatment
7	Can the surgery be performed using closed (arthroscopic) methods?	Treatment
8	How will the void left by the tumor be filled?	Treatment
9	Is bone cement harmful to the body?	Treatment
10	Can the tumor recur after surgery?	Follow-up
11	Is there a risk of a bone fracture (pathological fracture)?	Complications
12	Am I allowed to exercise or play sports?	Daily Living Activities
13	Can this turn into a malignant cancer over time?	Follow-up
14	Will this affect my child’s growth or height?	Complications
15	Do herbal treatments or specific diets help?	Daily Living Activities
16	How long and how often should I come for follow-up?	Follow-up
17	When can I return to work or school after surgery?	Daily Living Activities
18	Can similar tumors appear in other parts of my body?	Follow-up
19	Will I need physical therapy after the procedure?	Follow-up
20	Is this condition genetic, and will it pass to my children?	Diagnosis

Responses were generated using ChatGPT-5.2 (OpenAI) during query sessions conducted on February 5, 2026. To preserve ecological validity and reflect the way an unprompted layperson would most plausibly interact with the model, a deliberately minimalist, zero-shot prompting strategy was adopted. Each of the twenty questions listed in **Table 1** was entered verbatim, in English, exactly as formulated by the question-development panel, with no rephrasing, simplification, or translation. No system-level prompt, persona assignment (e.g., “act as an orthopedic oncologist”), audience specification (e.g., “explain to a patient”), output-format instruction (e.g., “cite peer-reviewed sources”), or contextual framing was provided. No clinical scenario, demographic context, or patient history was attached to any query.

Each question was submitted in an independent, freshly initiated chat session with no prior conversational history, ensuring that no answer could be influenced by context carried over from a preceding query. Each question was queried once; no regeneration, refinement, or follow-up prompting was performed, and the default model settings were used without modification of temperature or other generation parameters. The complete, unedited responses produced by the model were captured verbatim and used directly for quality evaluation.

Responses from ChatGPT-5.2 were evaluated by three independent, fellowship-trained orthopedic oncology surgeons — each with a minimum of five years of active clinical and surgical experience in orthopedic oncology, practicing at tertiary care referral centers — who were not involved in the question formulation process. During the evaluation, the quality of the responses was assessed using criteria adapted from the QAMAI approach, namely accuracy, clarity, relevance, comprehensiveness, source-basedness, and usability (**Table 2**). Evaluating specialists were instructed to scrutinize any references cited by the AI for existence and accuracy as part of the “provision of sources and references” criterion. Any response citing non-existent or unverifiable references was scored accordingly on that subdomain.

**Table 2:** Quality assessment criteria based on the QAMAI tool.

The quality analysis of medical artificial intelligence (QAMAI) tool
Accuracy: The information provided is accurate and up-to-date.
Clarity: The answer is clear and comprehensible in terms of language and scientific terminology.
Relevance: The information provided is relevant and directly answers the question posed.
Completeness: The response adequately covers all aspects of the question and provides sufficient information including areas of uncertainty.
Provision of sources and references: The response provides reliable sources and references to support the health information presented.
Usefulness: The response provides information sufficient to meet the user’s health information needs.

Responses were evaluated using a 5-point Likert scale following the original QAMAI scoring format,(14) in which raters indicate the degree to which they agree with a quality-related statement about each response, ranging from 1 (Strongly Disagree) to 5 (Strongly Agree) (**Table 3**).

**Table 3:** Five-point Likert scale used for response evaluation.

Score	Evaluation level	Description
1	Strongly Disagree	The response completely fails to meet this quality criterion.
2	Disagree	The response largely fails to meet this quality criterion.
3	Neither Agree nor Disagree	The response partially meets this quality criterion, with notable gaps.
4	Agree	The response largely meets this quality criterion, with only minor deficiencies.
5	Strongly Agree	The response fully and completely meets this quality criterion.

The total QAMAI score was calculated to be a maximum of 30 points. In statistical analyses, data were presented as mean and standard deviation. Inter-specialist agreement was assessed using the intraclass correlation coefficient (ICC), calculated using a two-way random effects model and absolute agreement approach. ICC values were interpreted according to commonly accepted thresholds: values below 0.50 were considered poor, between 0.50 and 0.75 moderate, between 0.75 and 0.90 good, and values above 0.90 were regarded as excellent agreement. This study does not require ethical committee approval as it does not contain patient data or personally identifiable information.

Statistical analyses were conducted using IBM SPSS Statistics for Windows, Version 26.0 (IBM Corp., Armonk, NY, USA). Continuous variables were expressed as mean ± standard deviation (SD). Inter-rater reliability was assessed using the ICC based on a two-way random-effects model with absolute agreement. A two-tailed p-value < 0.05 was considered statistically significant.

## 3 Results

### 3.1 Quantitative performance and domain analysis

**Figure 1:**
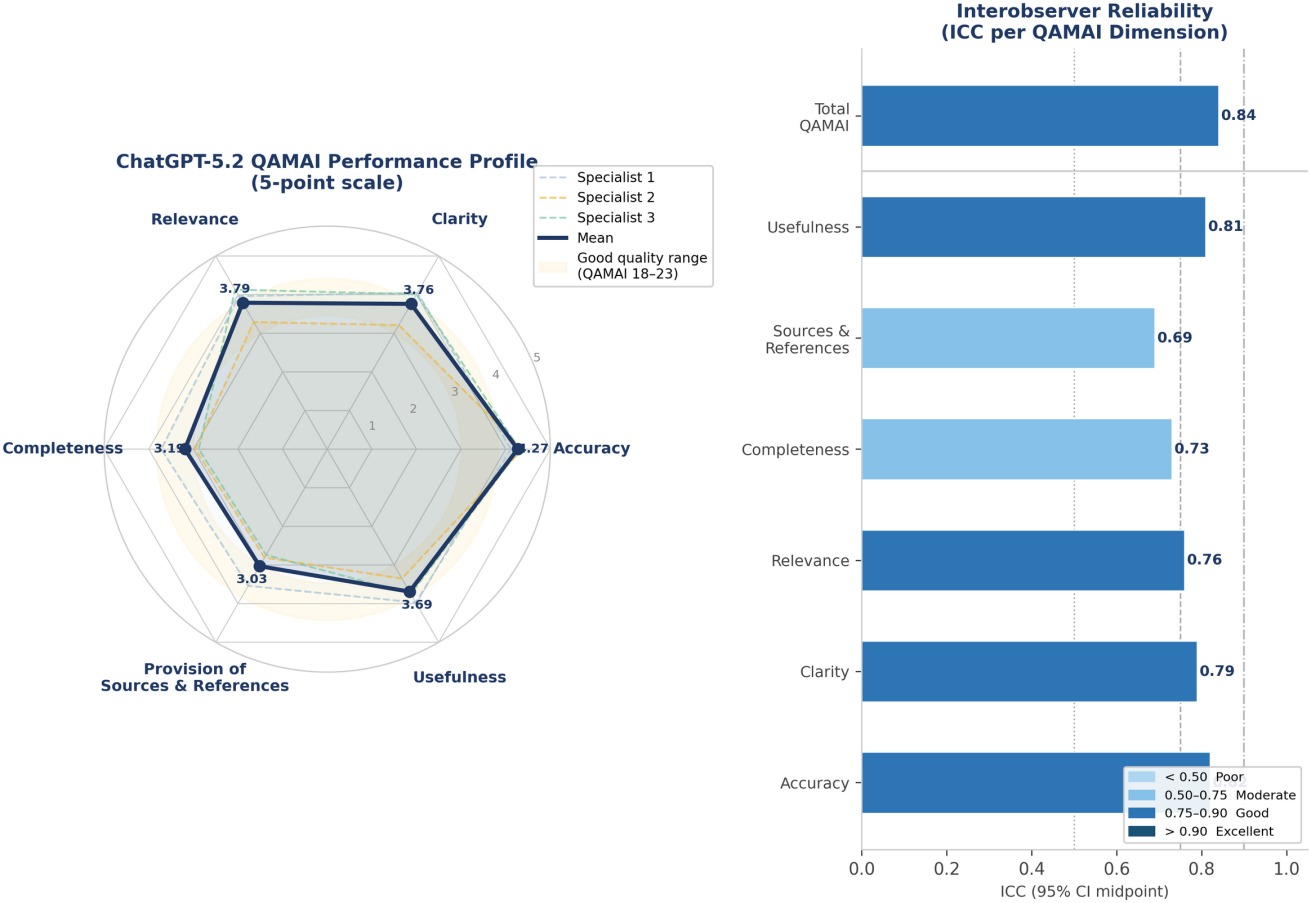
QAMAI evaluation results for ChatGPT-5.2 responses to benign bone tumor patient questions. Left: radar chart showing mean QAMAI scores across six evaluation dimensions for each specialist and overall mean (dark blue polygon). The shaded yellow band indicates the “good quality” range (total QAMAI score 18–23). Right: intraclass correlation coefficients (ICC) for each QAMAI subdomain and total score. ICC thresholds: <0.50 poor, 0.50–0.75 moderate, 0.75–0.90 good, >0.90 excellent.

The study utilized the QAMAI framework to evaluate ChatGPT-5.2 across six key dimensions using a five-point Likert scale. The model achieved an overall mean QAMAI score of 21.39 out of 30 (**Table 4**; **Figure 1**). According to the scoring thresholds established in the original QAMAI validation by Vaira et al. (14), a score of 18–23 corresponds to “good quality,” and the present result is therefore classified as good quality, reflecting a clinically satisfactory level of information for preliminary patient education. Among the specific subdomains, Accuracy was the highest-rated category with a mean score of 4.27. Conversely, the lowest scores were observed in Provision of sources and references (3.03) and Completeness (3.19).

**Table 4:** Specialist evaluation scores and interobserver reliability of chatbot responses according to QAMAI criteria.

QAMAI criteria	Spec. 1 (Avg ± SD)	Spec. 2 (Avg ± SD)	Spec. 3 (Avg ± SD)	Mean	ICC (95% CI)	Agreement level
Accuracy	4.12 ± 0.68	4.38 ± 0.32	4.31 ± 0.51	4.27	0.82 (0.71–0.90)	Good
Clarity	4.05 ± 0.72	3.21 ± 0.79	4.01 ± 0.48	3.76	0.79 (0.66–0.88)	Good
Relevance	3.96 ± 0.74	3.29 ± 0.76	4.13 ± 0.39	3.79	0.76 (0.62–0.86)	Good
Completeness	3.72 ± 0.81	2.96 ± 0.88	2.88 ± 0.69	3.19	0.73 (0.58–0.84)	Moderate
Sources & References	3.54 ± 0.83	2.81 ± 0.91	2.74 ± 0.49	3.03	0.69 (0.52–0.82)	Moderate
Usefulness	3.97 ± 0.70	3.35 ± 0.80	3.75 ± 0.71	3.69	0.81 (0.69–0.89)	Good
Total QAMAI (0–30)	23.36 ± 4.21	19.00 ± 4.47	21.82 ± 3.81	21.39	0.84 (0.74–0.91)	Good

### 3.2 Interobserver reliability and specialist variance

Three independent specialists evaluated all responses, yielding a total QAMAI ICC of 0.84 (95% CI: 0.74–0.91), classified as “good” agreement per the 0.75–0.90 threshold defined in the Methods section (**Table 4**; **Figure 1**). Subdomain ICC values ranged from moderate to good across all six QAMAI dimensions, confirming sufficient evaluator consistency for the purposes of this study.

### 3.3 Clinical significance

The findings suggest that while AI-based chatbots can play a vital supportive role in patient-physician communication, they possess inherent limitations that prevent them from replacing professional medical advice. High scores in Usefulness (3.69) and Relevance (3.79) indicate that these tools are helpful for addressing common patient anxieties regarding malignancy risks or surgery requirements. However, the model’s weakest area remains its limited access to current scientific literature and the potential for AI hallucinations in citations, which can lead to incomplete information if not carefully monitored.

## 4 Discussion

Many studies in the literature examine the answers given by chatbots to frequently asked questions by patients (15, 16). This research fills an important gap in the literature by being the first scientific study to systematically evaluate the quality of answers given by an artificial intelligence model to the most frequently asked questions by patients about benign bone tumors.

Benign bone tumors present a unique informational challenge in the context of AI-assisted patient education. Although non-malignant, a “tumor” diagnosis invariably generates substantial patient anxiety, and the questions most frequently raised in clinical practice — including fears about malignant transformation, pathological fracture risk, surgical necessity, genetic inheritance, and recurrence — are clinically nuanced and not uniformly addressed in general consumer health resources. In contrast to common orthopedic procedures, where patient information needs are relatively standardized, orthopedic oncology encompasses a heterogeneous group of lesions, each with distinct natural history, prognosis, and management considerations. This clinical heterogeneity means that accurate, lesion-specific, and evidence-based information is particularly important, and errors or omissions in AI-generated responses in this domain carry a heightened risk of patient misunderstanding or inappropriate health behavior. The findings of this study must therefore be interpreted not only in methodological terms but also against this specific clinical background.

A methodological consideration specific to this study is the approach used for question formulation. The clinician-derived methodology employed here — in which fellowship-trained orthopedic oncology specialists selected questions based on the concerns most frequently encountered in outpatient practice — is consistent with the approach adopted in the majority of comparable published studies in this field (17–24). This paradigm offers a key advantage in technically specialized domains: clinician-formulated questions are calibrated to clinically significant informational needs, ensuring that the AI responses evaluated address the issues that most directly affect patient understanding and safety. An alternative paradigm, used in a subset of studies in adjacent fields, involves deriving questions from population-level search behavior — for example, through Google-based search analysis (e.g., Google Trends keyword data or “People Also Ask” search results) — which may better capture lay terminology, patient anxiety-driven queries, and informational concerns that are underrepresented in clinical encounter data (25–27). These two approaches are not mutually exclusive; in the context of orthopedic oncology, the clinician-derived approach was considered more appropriate given the subspecialty complexity, where lay search behavior may not reliably reflect the clinically significant concerns that experienced specialists most commonly address. A comprehensive evaluation of AI performance in patient education for benign bone tumors would ideally triangulate both paradigms, and future studies combining clinician-formulated with empirically-derived patient queries would enhance representativeness and generalizability.

The selection of QAMAI as the sole evaluation instrument also warrants discussion. DISCERN, developed in the 1990s, was designed to assess the quality of written consumer health information in the form of leaflets, pamphlets, and web-based documents. Its core domains — source credibility, evidence basis, and balanced treatment option disclosure — are relevant to static informational materials but do not address characteristics specific to AI-generated conversational responses, such as response completeness within a single prompt, citation fabrication risk, or the coherence of multi-part clinical answers (28). Similarly, the EQIP tool was developed and validated for evaluating structured patient information pamphlets; its item structure assumes a fixed-format document and is therefore poorly suited to the open-ended, variable-length outputs produced by large language models (14). Readability indices such as the Flesch-Kincaid Grade Level, the SMOG index, and the Gunning Fog Score quantify linguistic complexity and reading grade level but do not capture factual accuracy, clinical completeness, or evidence quality; they address a fundamentally different dimension of response quality and cannot substitute for a content-validity instrument in a clinical evaluation context (29). In contrast, QAMAI was specifically developed and validated for the assessment of AI-generated medical responses and uniquely incorporates six dimensions directly relevant to chatbot output quality: accuracy, clarity, relevance, completeness, provision of sources and references, and usability (14). It is currently the only published and validated tool designed specifically for this purpose. The decision to rely solely on QAMAI, without supplementary readability or patient-comprehension instruments, is acknowledged as a limitation below.

The 88% accuracy rate in the Total Knee Replacement (TKR) study and the fact that at least 50% of the responses in the Anterior Cruciate Ligament (ACL) study were found to be “completely correct” are consistent with our findings, where the average accuracy score of ChatGPT was determined to be 4.27 (17, 18). Similarly, in the Unicondylar Knee Arthroplasty (UKA) study, Aydilek et al. reported that ChatGPT was 83.3% consistent with academic sources (19). This confirms that ChatGPT is successful in conveying basic medical information and definitions across different orthopedic subspecialties.

Beyond accuracy, the high Relevance (3.79) and Usability (3.69) scores are clinically significant findings that deserve explicit attention. These dimensions indicate that evaluating specialists consistently judged ChatGPT-5.2’s responses as contextually appropriate and as meeting the fundamental informational needs of patients — that is, the model demonstrably understood what each question was asking and provided a response that a physician would consider helpful as a starting point. This pattern is consistent with findings in adjacent musculoskeletal subspecialties. Kara et al., evaluating ChatGPT, Gemini, and Perplexity responses to frequently asked questions about ankylosing spondylitis, reported that AI-generated responses were generally reliable and appropriately structured for patient audiences, reflecting the model’s capacity to deliver domain-relevant content across musculoskeletal conditions requiring long-term patient engagement.(8) Similarly, Ayık et al., assessing ChatGPT responses to hip arthroscopy patient questions, reported generally adequate performance, while noting a quality decline for technically complex surgical queries — a pattern directly paralleled by the relatively lower completeness and source citation scores in the present study (30).

In the study by Anastasio et al., ChatGPT’s responses to foot and ankle pathologies were found to be “top-tier” quality by specialists at a rate of 68.2% (23). Smith et al. found a perfect agreement score of 3.0/3.0 in ankle ORIF scenarios with specialist opinion (24). These high rates are consistent with the present study’s relevance and usability findings, collectively indicating that ChatGPT’s capacity to provide contextually appropriate and patient-oriented responses is a reproducible characteristic across orthopedic subspecialties.

The comprehensiveness subdomain yielded the second-lowest score in this study (3.19), a finding that is consistent with the established pattern across the literature whereby AI models perform less well on complex, subspecialty-specific clinical questions than on general or post-procedural informational queries. Studies evaluating ChatGPT responses in domains characterized by well-standardized instructions — such as general orthopedic home care and early post-procedural guidance — have reported comparatively higher comprehensiveness scores, reflecting the model’s stronger performance in informational contexts that are broadly represented in its training data (31). However, because these studies employ different evaluation instruments, rating scales, and clinical contexts, direct numerical score comparisons with the present findings are not appropriate; this observation should therefore be understood as a qualitative contextual reference rather than a quantitative benchmark. The pattern is further supported within more methodologically comparable studies: in the Boxer fracture study by White et al., the AI scored “A-” on general questions but dropped to “C+” on management and technical questions, directly illustrating how clinical complexity suppresses comprehensiveness performance (20). Adelstein et al.’s evaluation of total shoulder arthroplasty (TSA) questions similarly returned a score indicating “requiring minimal or moderate explanation,” confirming that comprehensiveness deficits are a consistent feature of AI responses to complex surgical queries (21).

The lowest-scoring subdomain in this study was Provision of sources and references (3.03), a finding consistent with a well-documented limitation of large language models across clinical specialties. Hu et al. and Aydilek et al. both highlighted that restricted access to current peer-reviewed literature limits the quality of AI-generated scientific references in orthopedic contexts (19, 22). Walker et al. systematically compared ChatGPT responses against established clinical guidelines and patient information quality instruments, finding that while factual accuracy was generally adequate, citation reliability was inconsistent and frequently unverifiable — directly paralleling the present finding (15). Atik et al. specifically cautioned that ChatGPT should not be positioned as a replacement for healthcare professionals, emphasizing that AI-generated content lacking verifiable, evidence-based references poses a particular risk when patients use it as a basis for clinical decisions (11).

The mechanism underlying these citation deficits is “AI hallucination”, the generation of plausible-sounding but factually unsupported content, including non-existent references, fictitious DOIs, and misattributed findings. In clinical settings this constitutes a primary patient-safety concern. Kim et al. proposed a systematic taxonomy distinguishing factual fabrication, citation fabrication, reasoning hallucination, and contextual misalignment, and demonstrated that even state-of-the-art models continue to produce these errors at clinically meaningful rates (31). Empirical evidence supports this taxonomy: Bhattacharyya et al. found that 47% of references generated by ChatGPT in medical content were entirely fabricated and only 7% were both authentic and accurate, and Gravel et al. reported a 69% fabrication rate in references provided for medical questions (32, 33). The risk is heightened in orthopedic oncology, where patient queries frequently concern probabilistic outcomes such as malignant transformation rates, recurrence likelihood, and hereditary penetrance, in which a fabricated reference can lend false epistemic authority to an inaccurate estimate that patients cannot independently verify and may use to inform decisions about surveillance or surgical timing.

Several mitigation strategies have been proposed. Retrieval-augmented generation (RAG), in which model outputs are grounded in a curated corpus of peer-reviewed literature retrieved at inference time, has been shown in a recent systematic review to substantially reduce citation fabrication in medical applications (34); a representative clinical implementation is Almanac, which produced more accurate and safer responses than baseline GPT-4 when augmented with a curated vector database of clinical knowledge (35). Integration with structured knowledge bases such as PubMed, UpToDate, or society-issued clinical practice guidelines represents a complementary path that directly targets the completeness and source-citation deficits observed here. Specialist-tuned medical large language models offer a parallel line of defense; Med-PaLM, developed through medical-domain fine-tuning, was the first model to surpass the passing threshold on USMLE-style examinations (36). Tonmoy et al. additionally catalogue more than 30 hallucination-mitigation techniques, including prompt-level source-verification constraints and post-generation fact-checking pipelines (37). Until such architectures become standard in consumer-facing chatbots, AI-generated information in orthopedic oncology should be treated as a preliminary educational adjunct requiring physician verification of any citation, statistic, or prognostic claim.

The total QAMAI ICC of 0.84, classified as “good” agreement per the 0.75–0.90 threshold, is broadly consistent with interobserver values reported in comparable studies, including the TSA study (ICC = 0.859) and PJI study (ICC = 0.605) (21, 22). This consistency lends methodological credibility to the primary quality findings by confirming that the QAMAI scores reflect reproducible specialist judgments rather than evaluator-specific variation.

An important methodological consideration pertaining to the reproducibility of the reported findings is the stochastic nature of large language model outputs. Because each of the twenty questions was queried only once, in a single session, the QAMAI scores represent a point-in-time sample of ChatGPT-5.2’s output distribution rather than a stable benchmark. Large language models do not produce deterministic responses; sampling variability means that the same query submitted in a different session may yield a meaningfully different response in terms of content depth, specificity, and citation quality. Empirical studies specifically examining ChatGPT output consistency across repeated queries have confirmed this variability: Franc et al. found that only 49.3% of test cases yielded identical responses across ten repetitions, characterizing ChatGPT as exhibiting suboptimal repeatability and reproducibility (38), and Funk et al. reported that ChatGPT-4 produced consistent correct answers in only 78% of repeated queries of medical examination questions (39). This variability is likely most consequential for the “Provision of sources and references” criterion, where citation generation can fluctuate substantially between sessions, and for the “Completeness” criterion, where response length and clinical depth may vary. Although this single-query design is consistent with the methodology adopted in the majority of comparable published studies in this field, the reported subdomain scores should therefore be interpreted as approximations of typical model performance rather than as fixed values.

From a broader perspective, these findings carry important implications for the integration of AI chatbots into orthopedic oncology patient education. Clusmann et al. outlined a conceptual framework for the role of large language models in medicine, identifying their greatest potential as accessible first-contact informational tools that reduce informational asymmetry before specialist consultations, while emphasizing the continued necessity of physician oversight to ensure clinical safety and contextual accuracy (7). Castaneda et al. similarly argued that AI-based clinical decision support tools achieve optimal benefit when they serve as complements to — rather than substitutes for — physician guidance, supporting patient understanding without displacing the interpretive and communicative role of the clinician (10). The present findings align with this framework: the strong accuracy and usability performance of ChatGPT-5.2 supports its potential as a preparatory educational tool that helps patients with benign bone tumors formulate informed questions before outpatient consultations, understand basic diagnostic and treatment concepts, and manage anxiety through accessible preliminary information. However, the persistent deficiencies in completeness and citation quality underscore that AI-generated responses cannot substitute for individualized clinical guidance, particularly for prognosis, malignant transformation risk assessment, or surgical decision-making (40). Health systems and orthopedic oncology clinicians should therefore consider integrating AI-generated information as a structured adjunct to — but never a replacement for — patient-physician communication, with clear guidance provided to patients about the limitations of AI-generated health content (41, 42).

This study has several limitations. First, the evaluation reflects a single time-point assessment of ChatGPT-5.2; given the rapidly evolving nature of AI, results may differ with future model updates or alternative platforms. Second, each question was queried only once in a single session; given the stochastic nature of large language model outputs, the same prompt submitted in a different session could produce a response with different content depth, length, or citation composition, which limits reproducibility and may introduce random variance into individual QAMAI subdomain scores, particularly for “Provision of sources and references” and “Completeness.” While this design is consistent with comparable studies, future research employing multi-query protocols would yield more robust and reproducible score estimates. Third, a deliberately minimalist, zero-shot prompting strategy was used to reflect typical real-world patient–chatbot interactions; while this approach preserves ecological validity, it does not assess model performance under engineered or role-conditioned prompts, which may yield higher-quality responses and warrant evaluation in future studies. Fourth, the twenty questions were clinician-formulated rather than derived from empirical patient query data; while this approach prioritizes clinical accuracy and domain relevance, it may not capture the full breadth of real-world layperson concerns — including emotionally-driven queries and lay-media-sourced informational needs — as discussed in the Discussion section above. Fifth, the study focused exclusively on benign bone tumors, limiting generalizability. Sixth, scoring inherently involves subjective expert judgment, which may introduce evaluator-dependent variability despite good interobserver agreement. Seventh, the evaluation relied exclusively on QAMAI and did not incorporate complementary instruments such as readability indices (e.g., Flesch-Kincaid Grade Level) or patient-comprehension tools; while QAMAI captures clinical quality dimensions, the absence of readability assessment means that the suitability of AI-generated responses for patients with varying health literacy levels was not formally evaluated. Finally, the study did not investigate patient comprehension, behavioral impact, anxiety levels, or clinical outcomes. Future research should include comparative analyses across multiple AI models, multi-instrument evaluation frameworks incorporating readability measures, dynamic conversation simulations, systematic hallucination assessment, and patient-centered outcome measures.

## 5 Conclusion

This study demonstrated that AI responses to frequently asked questions about benign bone tumors were of good quality on the QAMAI scale, with good interobserver agreement among evaluating specialists (ICC = 0.84). High scores in accuracy and usability suggest that these responses can serve as a supportive tool in patient information processes. However, lower performance in source citation and completeness indicates that AI outputs should not be used in clinical practice without physician supervision. Overall, AI-based chatbots can play a supportive role in patient-physician communication in orthopedics but cannot replace clinical decision-making processes.

## Data Availability

The raw data supporting the conclusions of this article will be made available by the authors, without undue reservation.
